# Surface Engineering of Polymeric Colloidal Crystals by Temperature – Pressure Annealing

**DOI:** 10.1002/marc.202400668

**Published:** 2024-11-05

**Authors:** Jeena Varghese, Visnja Babacic, Mikolaj Pochylski, Jacek Gapinski, Hans‐Juergen Butt, George Fytas, Bartlomiej Graczykowski

**Affiliations:** ^1^ Faculty of Physics Adam Mickiewicz University Uniwersytetu Poznanskiego 2 Poznan 61–614 Poland; ^2^ Max Planck Institute for Polymer Research Ackermannweg 10 55128 Mainz Germany; ^3^ Institute of Electronic Structure and Laser F.O.R.T.H, N. Plastira 100 Heraklion 70013 Greece

**Keywords:** brillouin light scattering, polymer colloidal crystals, polystyrene nanoparticles, spheroidal lamb modes, temperature‐pressure annealing

## Abstract

Polymer colloidal crystals (PCCs) have been widely explored as acoustic and optical metamaterials and as templates for nanolithography. However, fabrication impurities and fragility of the self‐assembled structures are critical bottlenecks for the device's efficiency and applications. We have demonstrated that temperature‐assisted pressure [T,p] annealing results in the mechanical strengthening of PCCs, which improves with the annealing temperature. Here, the enhancement of elastic properties and morphological features of self‐assembled PCC's is evaluated using Brillouin light scattering and scanning electron microscopy. The pressure‐induced effects on the vibrational modes of PCCs are also illustrated at temperatures well below the polymer glass transition. While the PCCs colloid constituents display reversibility, the PCC material is strongly irreversible in the performed thermodynamic cycle. The effective elastic modulus enhances from 0.7 GPa for the pristine sample to 0.8 GPa, solely by pressure annealing at room temperature. [T,p] annealing at higher temperatures leads to a maximum effective elastic modulus of 1.7 GPa, more than twice the value in the pristine sample. Above a cross‐over pressure, $p_{c\;}(\approx$725 bar at 348 K), the PCCs respond elastically and, hence, reversibly to pressure changes.

## Graphical abstract

Polymer colloidal crystals have been widely explored as acoustic and optical metamaterials and as templates for nanolithography. However, fabrication impurities and fragility of the self‐assembled structures are critical bottlenecks for the device's efficiency and applications. This studydemonstrate that temperature‐assisted pressure [T,p] annealing results in the mechanical strengthening of PCCs, which improves with the annealing temperature.

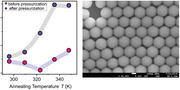

## 1 Introduction

Polymer nanoparticles self‐assembled into colloidal crystals (PCCs) form periodic structures that allow the controlled propagation of light and sound waves, realizing photonic^[^ ^1^, ^2^ ^]^ and phononic crystals,^[^ ^3^, ^4^, ^5^ ^]^ respectively. The void volume of 26% for the *fcc* crystal structure enables PCCs to be widely applied as sacrificial templates for creating patterned architectures^[^ ^6^ ^]^ and macroporous structures.^[^ ^7^, ^8^, ^9^ ^]^ However, the self‐assembled PCCs are brittle and can disintegrate quickly under external stress due to the weak van der Waals forces binding the constituent particles in the PCC.^[^ ^6^, ^10^ ^]^ The PCC's fragility can limit the devices' efficiency and, hence, the potential applications. Furthermore, it can result in micro‐ and nano‐contamination of the environment, which is another primary concern and can be life‐threatening.^[^ ^11^, ^12^, ^13^ ^]^ Therefore, the repeating unit cells of PCCs should be artificially engineered to generate desirable material properties. Among the several chemical and physical methods for mechanical strengthening of PCCs,^[^ ^10^, ^14^, ^15^, ^16^, ^17^, ^18^ ^]^ thermal annealing below the polymer glass transition $\left(T_{g}\right)$ is one of the most straightforward approaches. However, the process is relatively slow, and the enhancement of elastic modulus is not significantly high.^[^ ^10^ ^]^ Cold soldering by plasticization, when PCCs are exposed to high hydrostatic gas pressure, is a simple, cost‐effective, chemical‐free, and efficient method for the enhancement of elasticity even at room temperature conditions.^[^ ^10^, ^18^ ^]^ In contrast to the as prepared PCC's by drop casting of an aqueous suspension of the colloidal particles, applying pressure significantly strengthens the mechanical robustness of PCC, i.e., reduced detachment from the substrate.^[^ ^10^ ^]^

In this work, we demonstrate that temperature‐assisted – pressure [T,p] annealing of polystyrene (PS) colloidal crystals in the glassy phase enables mechanical reinforcement of the granular assembly that improves with the annealing temperature. As a model sample, we work on PCCs made of monodispersed PS nanoparticles (NPs) with a diameter of 268 nm. Nitrogen gas above the critical [T,p] condition, [126.2 K, 34 bar],^[^ ^19^ ^]^ attains the supercritical state and is a good solvent for polystyrene; it is used for high‐pressure annealing. Using Brillouin light scattering (BLS), we evaluate the trend of the GHz vibrational (Lamb) modes of the PCCs at the constant set temperature ranging from 296 to 348 K, well below the glass transition temperature of the bulk PS, $T_{g}\approx$375 K. The highest annealing temperature slightly exceeds the softening temperature, $\;T_{s}$ (= 343 K for PS268^[^ ^10^ ^]^) above which PS NP shell mobility occurs below $T_{g}$. The PCC material responds irreversibly to the [T,p] annealing cycle with an effective elastic modulus increasing with the annealing temperature up to an upper value of 1.7 GPa, from 0.7 GPa for the pristine sample. However, PCCs respond elastically and, hence, reversibly to pressure changes above a crossover pressure $p_{c\;}$ (= 725 bar at 348 K). Alternatively, the PS colloid constituents display reversibility for the performed thermodynamic cycle and reversible pressure dependence of the particle vibration above a pressure $p_{c\;}$ due to the closed PCC voids, a consequence of the enhanced particle contacts by compression and plasticization. Supercritical nitrogen, being a solvent for polystyrene, can effectively plasticize the PCC structure in addition to the elastic deformation by hydrostatic compression. Consequently, [T,p] annealing can enhance the effective elasticity of the PCC structure and can lower the glass transition temperature $T_{g}$.^[^ ^10^ ^]^

The paper is organized as follows: After the information on materials, experimental methods, and measurement protocol, the results are presented and discussed in the next section, with conclusions summarized at the end.

## 2 Experimental Section

### 2.1 Samples

Polystyrene (PS268) NPs of particle diameter d = 268 ± 7 nm were synthesized via surfactant‐free emulsion polymerization, as described elsewhere.^[^ ^20^, ^21^, ^22^ ^]^ Self‐assembled 3D PCCs were fabricated by drop‐casting the cleaned aqueous dispersion of PS NPs onto a pre‐cleaned glass substrate.^[^ ^10^, ^18^ ^]^ The samples were dried in a vacuum bell jar at room temperature (RT, 293 K) for a few hours before the BLS measurements. The thickness of the PCC samples deposited on the glass slides ranged from tens to hundreds of micrometers, enabling relatively fast BLS data acquisition. However, the same samples were unsuitable for SEM imaging due to the non‐conductive substrate and the possible impact of the electron beam on the contacts. For scanning electron microscope (SEM) (JEOL 8001TTLS system) images, the particle suspension was spin‐coated onto a pre‐cleaned silicon wafer at a spinning rate of 4000 rpm for 60 s and was vacuum dried before the measurements. Thus, thin spin‐coated NP layers were used on Si wafers for the SEM experiment. SEM images of PCCs, before and after [T,p] annealing process, are presented in Figure 2 below. Structural information of the BLS samples could, however, be inferred by optical microscopy and imaging scan by SEM along the cross‐section of the BLS samples.^[^ ^23^ ^]^ The SEM image of (3D drop casted) as‐prepared PS268 CC sample showing the crystalline arrangement of NPs is shown in Figure S1 (Supporting Information).

### 2.2 Brillouin Light Scattering

Spontaneous Brillouin light scattering (BLS) is a powerful, non‐destructive optical tool based on inelastic light scattering by thermally activated hypersonic phonons in the material. The angular frequency of an acoustic phonon is given by ω=2πf. For a given scattering geometry, the scattering wave vector (q) is defined as $q=\pm (k_{i}\;-\;k_{s})$, where $k_{i}$ and $k_{s}$ denote the wave vectors of incident and scattered light, respectively. Generally, the magnitude of the scattering (phonon) wave vector in the medium is given by $q=\left(\frac{4\pi n}{\lambda }\right)\;\sin\left(\frac{\theta }{2}\right)$, where n is the material's refractive index, λ the wavelength of the probing laser beam, and θ is the scattering angle between the incident $\left(k_{i}\right)$ and scattered $\left(k_{s}\right)$ wave vectors. For dry PCCs, q is ill‐defined due to the multiple light scattering in the material.^[^ ^24^ ^]^ Consequently, BLS spectrum resolves the collective vibrations of the PS NPs in the CC structure, determined by the material's elastic properties and scattering geometry. BLS records the particle vibrations which are localized and therefore independent of the scattering wave vector.

### 2.3 Temperature – Pressure Dependent in‐Situ Brillouin Spectroscopy of PCCs

A high‐pressure experimental setup consisting of a custom‐made steel chamber, N_2_ gas cylinder (200 bar), and a gas compressor with a pressure gauge was used.^[^ ^10^ ^]^ The as‐fabricated PCC samples were fixed in a cylindrical metallic cuvette and placed inside the high‐pressure chamber. The N_2_ gas was supplied at constant set pressures to the chamber through a metallic pipeline. The pressure was applied directly from the gas bottle up to 200 bar, and a compressor was used to increase the pressure to 1000 bar. The BLS experiments were performed in the backscattering (BS) geometry to obtain the maximum scattered light intensity.^[^ ^25^, ^26^ ^]^ A continuous wave green laser (COHERENT Verdi 5) of wavelength λ=532 nm was used as the probing light source, and the beam was focused using a lens (focal length 125 mm) on the sample through the pressure chamber's quartz window. The set laser power was 400 mW. To suppress the BLS signals originating from the acoustic phonons of gas, a cross‐polarized configuration was used where the incident and scattered laser beam were of vertical and horizontal (V‐H) polarizations, respectively. A polarizing cube beam splitter (Thorlabs, PSB103) was used to achieve the cross‐polarized configuration, as it transmits horizontally and reflects a vertically polarized beam. The scattered laser beam was directed toward a high‐contrast six‐pass tandem Fabry‐Perot interferometer (JRS Optical Instruments) to detect the vibrational spectra of the NPs. The acoustic impedance measurements of the N_2_ gas were estimated from the longitudinal acoustic phonon (pressure wave) velocity.^[^ ^18^ ^]^ Temperature control was achieved using a circulator Bath Chiller (HAAKE K35) that allows temperature control from −35 to +200 °C. The circulator is connected to the sample chamber through specific valves, and a thermocouple could determine the temperature inside the chamber. The N_2_ gas pressure was applied to the as‐prepared PS268 CC samples at five different temperatures, T, between 296 K (room temperature, RT) to 348 K. At ambient conditions, PS colloidal particles exhibit limited contacts compared to the post‐treated PS CCs elevated pressures and temperatures as inferred by SEM in Figure 2.

## 3 Results and Discussion

### 3.1 Temperature‐Pressure Annealing

**Figure 1:**
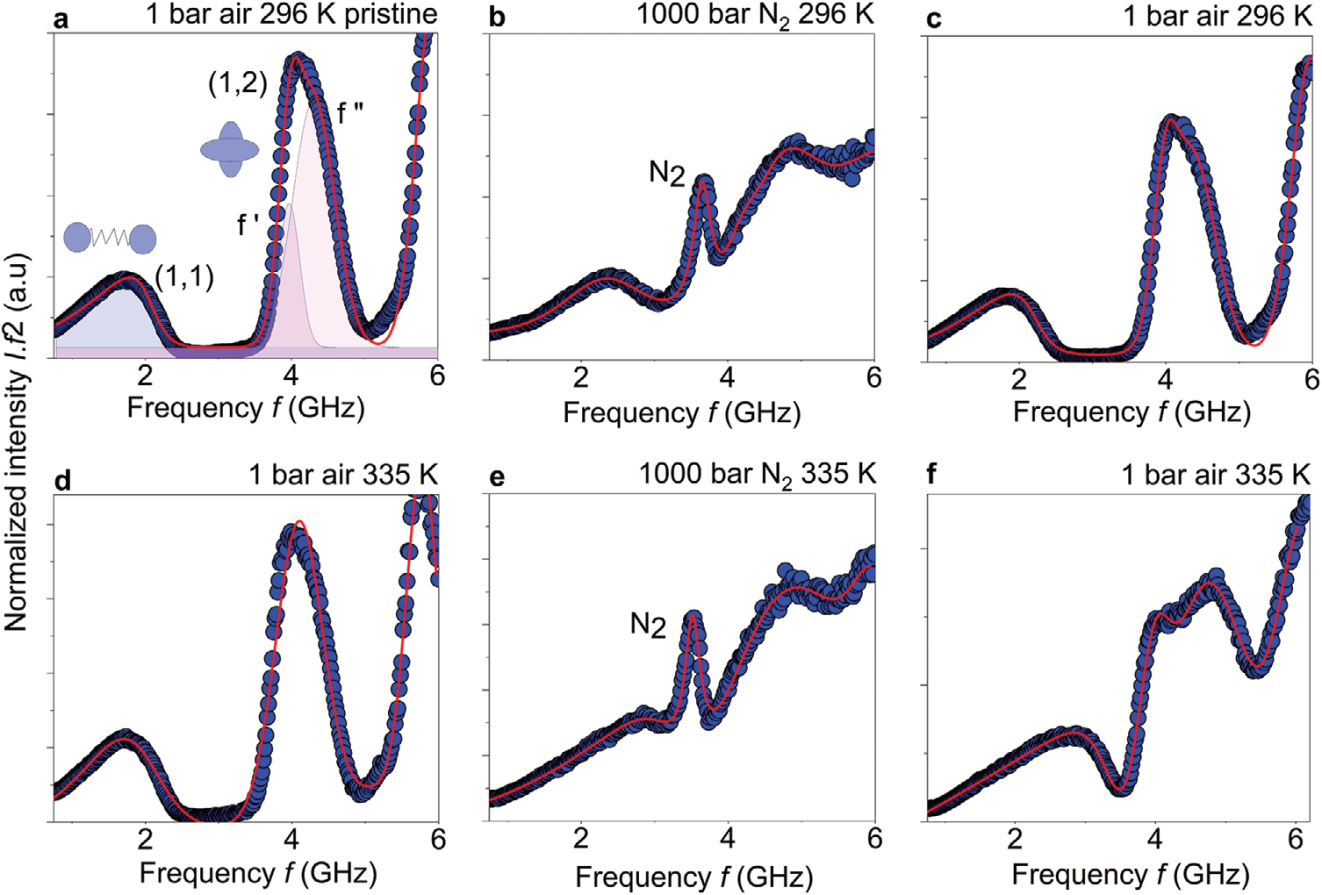
Exemplary BLS power spectra (blue solid circles, anti‐Stokes side) a–f) of PS268 (d= 268 nm) CCs (rescaled normalized intensity axis). BLS spectra measured at room temperature (296 K); the pristine sample at atmospheric pressure (1 bar of air) a), at 1000 bar of N_2_ b), and after depressurization at 1 bar of air c). d–f) BLS spectra as for a–c) but at a higher temperature of 335 K. The red solid lines indicate the fits of the BLS experimental data (blue solid circles) by Asym2sig and double Gaussian functions for the dipolar (1,1) and quadrupolar (1,2) spheroidal Lamb modes, respectively. The sharp peak shown in the BLS spectra of b,e) corresponds to the longitudinal phonon propagating in N_2_ gas at 1000 bar.

We examined the effect of temperature‐assisted – pressure [T,p] annealing on the elastic properties of PCCs selecting temperatures lower than the bulk PS glass transition temperature $T_{g}\approx 375\;K$. Among these temperatures, the lowest (296 K) and the highest (348 K) are, respectively, the ambient (RT) and the temperature slightly above the PS surface softening temperature, $\;T_{s}$ (= 343 K for PS268^[^ ^10^ ^]^). The latter defines the temperature at which the PS shell becomes mobile below $T_{g}$. **Figure** **1** displays exemplary normalized (by the phonon thermal population factor $\propto 1/f^{2}$)^[^ ^22^ ^]^ BLS spectra (rescaled reduced intensity axis) of PCCs that were recorded at two thermal conditions: at room temperature (RT, 296 K) (Figure 1a–c) and at 335 K (Figure 1d–f). The BLS spectra during the [T,p] annealing process were recorded for 2 min at different pressures from 1 to 1000 bar, changing by fixed steps (25 bars) at the constant temperature.

The spectrum shown in Figure 1a consists of two asymmetric peaks assigned to the dipolar (1,1) and quadrupolar (1,2) spheroidal Lamb modes. The dipolar mode is also referred to as the interaction‐induced mode, the frequency of which can indirectly measure the interfacial connectivity (contact radius, $a_{0}$) between the particles by using the Johnson‐Kendall‐Roberts (JKR) theory.^[^ ^27^, ^28^, ^29^ ^]^ The (1,1) mode is fitted with Asym2sig function^[^ ^30^ ^]^ to estimate the frequency $f_{1,1},$ which can yield the longitudinal phonon velocity in the *fcc* crystal along the [100] direction, $v_{\left[100\right]}=\pi df_{11}/\sqrt{2}$ and thereby the effective elastic constant of the PCCs:

(1) $$E_{\mathrm{eff}}=\rho_{\mathrm{eff}}v_{\left[100\right]}^{2}=\frac{1}{2}\rho_{\mathrm{eff}}\pi^{2}d^{2}f_{11}^{2},$$

The effective mass density of the *fcc* colloidal assembly, given the particle volume fraction as 0.74, $\rho_{\mathrm{eff}}=0.74\;\rho_{\mathrm{PS}}+\left(1-0.74\right)\;\rho_{\mathrm{air}}$, amounts to $777\;\mathrm{kg}\;m^{-3}$, with the mass densities of PS, $\rho_{\mathrm{PS}}=1050\;\mathrm{kg}\;m^{-3}$ and air, $\rho_{\mathrm{air}}=1.2\;\mathrm{kg}\;m^{-3}$ at ambient conditions (1 bar and 293 K).

First, we examine the [T,p] annealing effect on the spectral position and line shape of the interaction–induced – dipolar (1,1) mode in the BLS spectra (Figure 1a–f). Comparing the pristine sample at ambient conditions (1 bar air, 296 K, Figure 1a) to the temperature‐treated sample (1 bar air, 335 K, Figure 1d), we can infer that $f_{1,1}$ shows a slight red shift (≈1%) upon a temperature increase from 296 to 335 K. The decrease of $f_{1,1}$ can be understood as the thermal softening of the PS NPs.^[^ ^10^ ^]^ On applying 1000 bar N_2_ gas pressure at RT (296 K), $f_{1,1}$ of the pristine sample increases from 1.61 GHz (Figure 1a) to 2.35 GHz (Figure 1b), exhibiting ≈46% frequency blue shift. This further increases to 2.82 GHz at 335 K, amounting to a 75% frequency blue shift. Upon post‐pressure treatment, $f_{1,1}$ returns to 1.70 GHz at RT and 2.49 GHz at 335 K, assuming a 5.6% and 55% blue shift compared to the initial $f_{1,1}$ of the pristine samples at these two temperatures. The blue‐shift of the dipolar (1,1) mode after p annealing is more pronounced at 335 K than at 296 K.

The irreversibility of the BLS spectra after [T,p] annealing implies enhanced particle connectivity and, thereby, mechanical strengthening of PCCs triggered by gas permeation through the PS colloidal surface.^[^ ^10^ ^]^ Supercritical nitrogen is a good solvent for polystyrene (*H* = 0.087 × 10^−5 ^molg^−1^bar^−1^ at 298 K below $T_{g}$),^[^ ^10^, ^31^ ^]^ which can induce plasticization of the PS NP surface and thereby the mechanical strengthening of PCCs. Typically, the solubility of gas in fluids decreases with increasing temperature.^[^ ^32^, ^33^ ^]^ However, in the case of N_2_, a reverse solubility in glassy PS is reported, which saturates at T > 353 K;^[^ ^34^ ^]^ note that this observation is in contrast to Henry Law's in liquids. Therefore, N_2_ pressure annealing results in an irreversible change of the PCCs structure, with the irreversibility becoming stronger with the annealing temperature from RT to 348 K.

**Figure 2:**
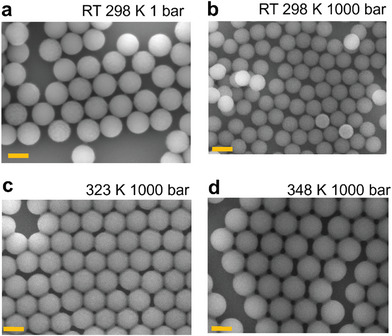
PS268 CC samples spin‐coated on silicon wafer substrates with a spinning rate of 4000 rpm over 60 s; pristine sample at 298 K, 1 bar air a), and after pressure treatment at 1000 bar N_2_ at different temperature conditions 298 K b), 323 K c) and 348 K d). T−P annealing displays enhanced particle contacts. The scale bars are 200 nm.

Turning to the high‐frequency quadrupolar (1,2) mode, its frequency relates to the elastic properties of the material and the interparticle interactions in the PS particle assembly. The particle contacts lower the NP's spherical symmetry, which leads to the spectral split of the (1,2) mode.^[^ ^22^ ^]^ Hence, quadrupole mode is fitted by double Gaussian line shapes centered at $f^{\prime }$ and $f^{\prime \prime }$ as given in Figure 1a. The reduced frequency, $f_{1,2}=2f^{\prime }-f^{\prime \prime }$, approximates the frequency of (1,2) mode of an interaction – free single particle, which can be used to determine the particle diameter by.^[^ ^1^, ^4^ ^]^

(2) $$f_{1,2}=Av_{t}/d$$

where A ≃ 0.84 for materials with Poisson's ratio in the range of 0.16–0.32^[^ ^35^ ^]^ and $v_{t}$ = 1210 m s^−1^ for bulk PS. For the pristine sample at ambient conditions (Figure 1a), $f_{1,2}$ = 3.63 GHz ($f^{\prime }$ = 3.95 GHz, $f^{\prime \prime }$ = 4.28 GHz) estimates the particle diameter to be 280 nm; using PS bulk elasticity, d in Equation (2) exceeds the corresponding SEM value (268 nm) by ≈4%. This slight disparity can probably arise from a small elasticity variation of PS because of the robust spherical shape (**Figure** **2**a). Increasing the temperature to 335 K (Figure 1d), $f_{1,2}$ = 3.53 GHz, and the moderate redshift is attributed to the thermal softening of PS. However, when applying N_2_ gas pressure at 1000 bar_,_ $f_{1,2}$ (= 3.87 GHz) displays ≈7% blue shift at RT (Figure 1b), while it displays pristine–like behavior at 335 K ($f_{1,2}$ = 3.62 GHz, Figure 1e). After depressurization at 1 bar air, $f_{1,2}$ = 3.60 GHz at RT (Figure 1c), indicating complete reversibility, and 3.37 GHz (≈7% redshift) at 335 K (Figure 1f). Notably, $f_{1,2}$ frequency range seems to be robust to the [T,p] annealing process that might suggest reversible behaviour for the PS colloid constituent in contrast to the PCCs material manifested in the dipolar $\;f_{1,1}$ (previous paragraph). However, the enhanced particle contacts on the [T,p] annealing in the case of (1,1) mode is also manifested in the prominent broadening and spectral split (Figure 1f) of the peak corresponding to the quadrupolar (1,2) mode. It is evident that the spectra are poorly resolved at 1000 bar N_2_ for 296 K (Figure 1b), and 335 K (Figure 1e), which is due to the mode delocalization as a result of the acoustic energy leakage from the particles to the voids. The acoustic impedance contrast Z is lowered between PS and N_2_ with increasing pressure, and it reaches $\frac{Z_{\mathrm{PS}}}{Z_{N2}}$ = 4.7 at 1000 bar (Figure S2, Supporting Information).

The expected contact radius $a_{0}$ for PS NPs of 268 nm based on JKR theory (no external load, for weak van der Waals interactions between NPs)^[^ ^27^, ^28^ ^]^ can be calculated by:

(3) $$a_{0}^{\mathrm{JKR}}={\left(\frac{3\pi d^{2}W_{\mathrm{PS}-\mathrm{PS}}}{8E_{\mathrm{PS}\left(\mathrm{eff}\right)}}\right)}^{\frac{1}{3}}\;$$

Using d = 268 nm, adhesion energy, $W_{\mathrm{PS}-\mathrm{PS}}$ = 0.0636 Jm^−2^ and PS effective Young modulus, $E_{\mathrm{PS}(\mathrm{eff})}$ = 3.05 GPa,^[^ ^5^, ^10^, ^22^, ^36^ ^]^ Equation (3) yields $a_{0}\approx$ 12 nm. However, $a_{0}$ obtained from BLS data for the as‐prepared sample is 30 nm (using $a_{0}^{\mathrm{JKR}}=\frac{5\pi^{2}Mf_{11}^{2}}{9E_{\mathrm{PS}(\mathrm{eff})}}\;,$ derivation in Supporting Information). This discrepancy indicates that the particles are bonded by fabrication impurities in addition to the van der Waals interactions.^[^ ^18^, ^37^, ^38^ ^]^

The SEM images of the as‐prepared sample at ambient conditions (1 bar air, 298 K) suggest that the shape of the PS NPs is robust with a diameter d=268±7 nm. However, the size obtained from BLS is ≈4% higher due to a small variation of PS elasticity from its bulk state. The morphological changes of PCCs after applying pressure at elevated temperatures revealed by the SEM images in Figure 2b–d indicate that the spherical particle assembly is modified into a nearly hexagonal shape. Enhanced contacts and precise close‐packed assembly are observed after treatment at 323 K (Figure 2c) and 348 K (Figure 2d) at 1000 bar N_2_.

### 3.2 Component Versus Material Annealing

**Figure 3:**
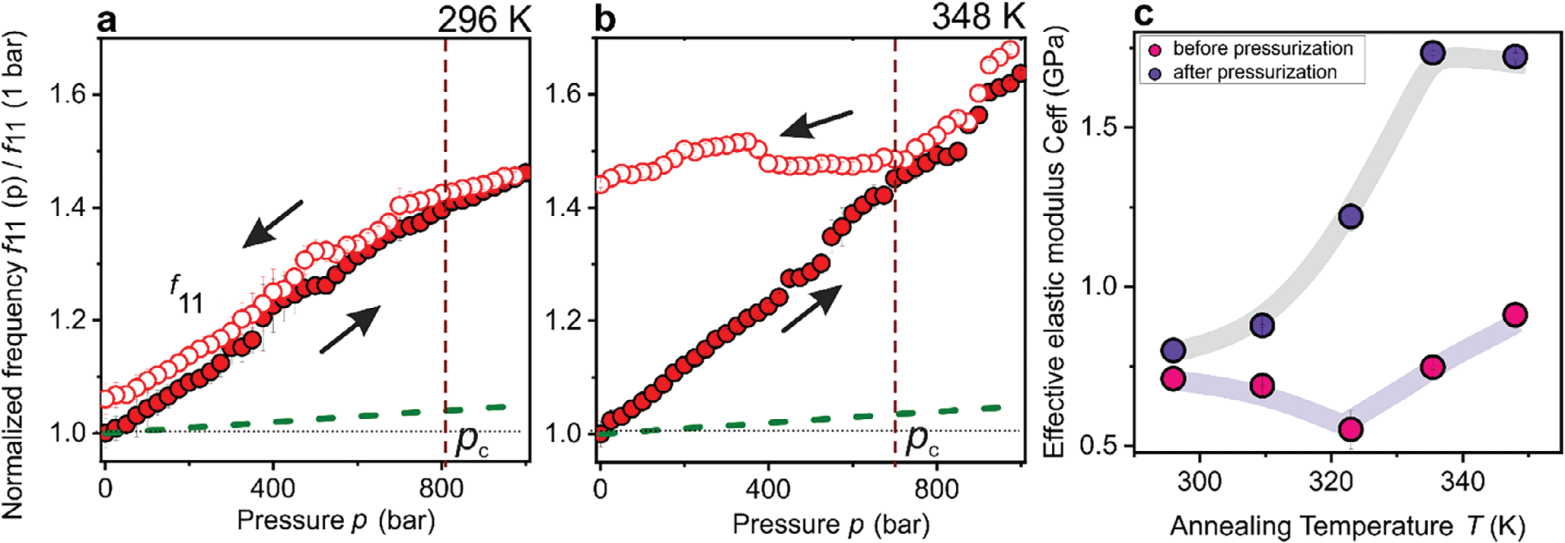
Normalized frequency $f_{11}^{*}\;\left(p\right)=$ $f_{11}\left(p\right)/f_{11}\left(1\;\mathrm{bar},\;T\right)$ of the dipolar (1,1) mode as a function of N_2_ pressure increase (red solid circles) and decrease (hollow circles) measured for PS268 CCs at specific temperatures, 296 K (RT) a), 348 K b). Dashed green lines in a,b) represent the trend calculated considering only the nonlinear stiffening of polystyrene with pressure increase. c) Effective elastic modulus $E_{\mathrm{eff}}$ as a function of annealing temperature obtained before (pink solid circles) and after (purple solid circles) pressure annealing.

**Figure 4:**
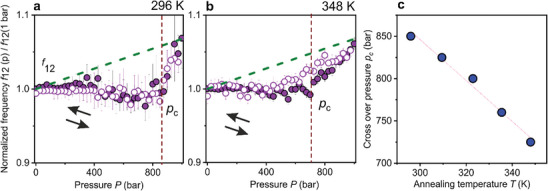
Normalized frequency $f_{12}^{*}\;\left(p\right)=$ $f_{12}\left(p\right)/f_{12}\left(1\;\mathrm{bar},\;T\right)$ of the quadrupolar (1,2) mode as a function of N_2_ pressure increase (purple solid circles) and decrease (hollow circles) measured for PS268 CCs at temperatures of 296 K (RT) a), 348 K b). Dashed green lines in a,b) represent the trend calculated considering only the nonlinear stiffening of polystyrene with pressure increase. c) Crossover pressure $p_{c}$ (blue solid circles) at different annealing temperatures.

The qualitative description of PCCs phase states upon [T,p] annealing is supported by the quantitative spectral analysis that reveals additional information on the thermodynamic cyclic process, discussed next in terms of the evolution $f_{11}\left(T,p\right)$ and $f_{12}\left(T,\;p\right)$ in **Figure** **3** and **Figure** **4**, respectively. The normalized frequency $f_{11}^{*}\;\left(p\right)=$ $f_{11}\left(p\right)/f_{11}\left(1\;\mathrm{bar},\;T\right)$ of the experimental dipolar frequency $f_{11}\left(p\right)$ to the corresponding value $f_{11}\left(1\;\mathrm{bar},\;T\right)$ at ambient pressure condition is shown as a function of increasing (solid red circles) and decreasing (hollow red circles) N_2_ pressures at two selected temperatures in Figure 3a,b; the temperatures include ambient 296 and 348 K ($T_{s}$ < 348 K < $\;T_{g}$). The dashed green lines in Figure 3a,b represent the simulated trend of $f_{11}\left(p\right)$ only considering the nonlinear stiffening of glassy PS. The increase of $f_{11}$ (p) is much stronger than anticipated by compression of PS (dashed line) in the glassy state, implying a consolidation due to surface contacts enabled by N_2_ plasticization of the PS colloids. However, the increased contact density at 296 K is almost reversible, as indicated by its upward (solid) and downward (open symbols) and close to the pristine CCs state at 1 bar (see also Figure 1a). SEM images of Figure 2 a,b have also confirmed the conserved spherical shape of PS colloids with some contacts after depressurization at 1 bar air. Pressurization at elevated temperatures (Figure 3b) renders the cyclic process irreversible, as is evident by the elevated value of $f_{11}$ (≈1.4 times higher than the 1 bar, 348 K state) returning to the initial state at 1 bar. The prominent increase of $f_{11}$ (up to 1.7 times at 1000 bar than the initial state) is attributed to the increased particle contact by compression and plasticization, in addition to the nonlinear stiffening of PS. On decreasing pressure, pressure‐induced stiffening is reduced. However, plasticization still occurs, modifying the PS surface. A close inspection of Figure 3b reveals that the process is reversible from 1000 bar down to a pressure 𝑝_c_ (≈700 bar at 348 K), and the PCCs respond elastically to pressure changes above 𝑝_c._

Figure 3c illustrates the effective elastic modulus as a function of the annealing temperature before (pink solid circles) and after (purple solid circles) p annealing. The effective elastic modulus, $E_{\mathrm{eff}}$, (Equation 1) of the pristine sample at 1 bar, 296 K is 0.7 GPa. On increasing the temperature up to 323 K without p annealing, $E_{\mathrm{eff}}$ first decreases with the annealing T due to the thermal softening of PS. However, $E_{\mathrm{eff}}$ increases above 323 K, reaching 0.9 GPa (28% improvement by thermal annealing) at 348 K. On the other hand, $E_{\mathrm{eff}}$ should be 0.8 GPa (14% improvement) solely by p treatment at room temperature. With T‐annealing accompanied by p treatment, $E_{\mathrm{eff}}$ is significantly enhanced, reaching an upper value of 1.7 GPa (142% improvement) saturating above T=335K, close to $\;T_{s}$. The constant value of $E_{\mathrm{eff}}$ above 335 K suggests that the voids are completely closed (no available free volume) due to the accelerated plasticization of PS NPs. The latter results from the increased solubility of N_2_ in PCCs and it saturates at high temperatures.^[^ ^18^ ^]^ While Figure 3 demonstrates the irreversible transformation of the PCCs films, possible alterations of the constituent PS colloids elasticity with [T,p] annealing are examined next in terms of Figure 4.

Figure 4a,b shows the normalized quadrupolar frequency, $f_{12}^{*}\;\left(p\right)=$ $f_{12}\left(p\right)/f_{12}\left(1\;\mathrm{bar},\;T\right)$ of the experimental $f_{12}\left(p\right)$ to the corresponding $f_{12}\left(1\;\mathrm{bar},\;T\right)$ at 1 bar conditions as a function of increasing (solid purple circles) and decreasing (hollow purple circles) N_2_ pressures at 296, and 348 K. The green dashed lines denote the theoretical trend based solely on the elastic deformation of PS by hydrostatic compression. At first, the reduced $f_{12}$ (Figure 4a,b) follows the predicted trend up to ≈250 bar for both temperatures, as $f_{12}$ increases by hydrostatic compression. Later on, $f_{12}$ slightly decreases with pressure due to increased acoustic energy leakage from the PS NPs to the voids. The experimental $f_{12}$ is lower than the predicted trend based only on the compression of glassy PS (green dashed lines) due to the increased acoustic energy leakage with pressure ($Z_{\mathrm{PS}}/Z_{N2}$ at different pressures is given in Figure S2 (Supporting Information)^[^ ^18^ ^]^. The initial decrease of $f_{12}$ with p reverses at a crossover pressure $p_{c}$, above which the acoustic energy leakage is considerably reduced due to the closing of voids (no available free volume), resulting in the enhancement of reduced $f_{12}$. The effect of enhanced particle contacts and stiffening by compression and plasticization is prominent here.^[^ ^18^ ^]^ The reduced $f_{12}$ follows a reversible path at 296 K (Figure 4a) and 348 K (> $T_{s}$) (Figure 4b). The crossover $p_{c}$ is observed at ≈850 bar in the case of p‐ annealing at 296 K (RT) (Figure 4a). Notably, $p_{c}$ decreases with annealing T (Figure 4c), due to enhanced particle soldering and closing of voids with [T,p] annealing due to the increased solubility of N_2_ at higher temperatures.

### 3.3 Concluding Remarks

Polymer colloidal crystals (PCCs) are periodic structures created by the self‐assembly of polymer particles. To tackle the fragility of self‐assembled PCCs, we have demonstrated a simple, uniform, and low‐cost laboratory method based on temperature‐assisted pressure [T,p] annealing.^[^ ^10^ ^]^ In this work, we examine the effect of pressure annealing at constant set temperatures by exposing PCCs of PS spheres with a diameter of 268 nm to supercritical N_2_. Using Brillouin light scattering, we evaluate the vibrational modes of the PS NPs and the characteristic trend of the quadrupolar particle and dipolar PCC vibrational frequencies in situ with the pressure‐temperature changes. The behavior of vibrational frequencies demonstrates the competitive effects of an increase of particle contacts by plasticization, nonlinear stiffening by hydrostatic compression, and acoustic energy leakage. However, [T,p] annealing results in an irreversible modification of PS CCs, which is evident in the enhancement of the elastic properties of the sample. The effective elastic modulus $E_{\mathrm{eff}}$ increases with an increase in temperature, reaching a maximum (≈1.7 GPa) in the vicinity of the softening temperature (≈343 K) of the PS268 surface. [T,p] annealing significantly improves the mechanical robustness of fragile PCCs, as evidenced by the decreased detachment of the fragile CCs from the substrate during the resilience test^[^ ^10^ ^]^ (Figure S4, Supporting Information).

## Conflict of Interest

The authors declare no conflict of interest.

## Supporting information



Supporting Information

## Data Availability

The data that support the findings of this study are available from the corresponding author upon reasonable request.

## References

[1] A. V. Akimov , Y. Tanaka , A. B. Pevtsov , S. F. Kaplan , V. G. Golubev , S. Tamura , D. R. Yakovlev , M. Bayer , Phys. Rev. Lett. 2008, 101, 033902. 18764257 10.1103/PhysRevLett.101.033902

[2] Z. Cai , Z. Li , S. Ravaine , M. He , Y. Song , Y. Yin , H. Zheng , J. Teng , A. Zhang , Chem. Soc. Rev. 2021, 50, 5898. 34027954 10.1039/d0cs00706d

[3] T. Vasileiadis , J. Varghese , V. Babacic , J. Gomis‐Bresco , D. Navarro Urrios , B. Graczykowski , J. Appl. Phys. 2021, 129, 160901.

[4] E. Alonso‐Redondo , M. Schmitt , Z. Urbach , C. M. Hui , R. Sainidou , P. Rembert , K. Matyjaszewski , M. R. Bockstaller , G. Fytas , Nat. Commun. 2015, 6, 8309. 26390851 10.1038/ncomms9309 PMC4595630

[5] B. Graczykowski , N. Vogel , K. Bley , H.‐J. Butt , G. Fytas , Nano Lett. 2020, 20, 1883. 32017578 10.1021/acs.nanolett.9b05101 PMC7068716

[6] H. Cong , B. Yu , J. Tang , Z. Li , X. Liu , Chem. Soc. Rev. 2013, 42, 7774. 23836297 10.1039/c3cs60078e

[7] H. Cong , B. Yu , J. Colloid Interface Sci. 2011, 353, 131. 20926097 10.1016/j.jcis.2010.09.040

[8] H. Cong , W. Cao , Solid State Sci. 2006, 8, 1056.

[9] X. S. Zhao , F. Su , Q. Yan , W. Guo , X. Y. Bao , L. Lv , Z. Zhou , J. Mater. Chem. 2006, 16, 637.

[10] V. Babacic , J. Varghese , E. Coy , E. Kang , M. Pochylski , J. Gapinski , G. Fytas , B. Graczykowski , J. Colloid Interface Sci. 2020, 579, 786. 32673855 10.1016/j.jcis.2020.06.104

[11] J. Liu , Y. Ma , D. Zhu , T. Xia , Y. Qi , Y. Yao , X. Guo , R. Ji , W. Chen , Environ. Sci. Technol. 2018, 52, 2677. 29420017 10.1021/acs.est.7b05211

[12] Y. Chae , Y.‐J. An , Mar. Pollut. Bull. 2017, 124, 624. 28222864 10.1016/j.marpolbul.2017.01.070

[13] E. Besseling , B. Wang , M. Lürling , A. A. Koelmans , Environ. Sci. Technol. 2014, 48, 12336. 25268330 10.1021/es503001d PMC6863593

[14] B. You , N. Wen , L. Shi , L. Wu , J. Zi , J. Mater. Chem. 2009, 19, 3594.

[15] P. Wang , B. Sun , T. Yao , M. Chen , X. Fan , H. Han , L. Li , C. Wang , Chem. Eng. J. 2017, 326, 1066.

[16] Y. Zhang , X. Hao , J. Zhou , Y. Zhang , J. Wang , Y. Song , L. Jiang , Macromol. Rapid Commun. 2010, 31, 2115. 21567638 10.1002/marc.201000495

[17] L. Wegewitz , A. Prowald , J. Meuthen , S. Dahle , O. Höfft , F. Endres , Phys. Chem. Chem. Phys. 2014, 16, 18261. 25058172 10.1039/c4cp01932f

[18] J. Varghese , R. Mohammadi , M. Pochylski , V. Babacic , J. Gapinski , N. Vogel , H.‐J. Butt , G. Fytas , B. Graczykowski , J. Colloid Interface Sci. 2023, 633, 314. 36459936 10.1016/j.jcis.2022.11.090

[19] I. D. Mantilla , D. E. Cristancho , S. Ejaz , K. R. Hall , M. Atilhan , G. A. Iglesias‐Silva , J. Chem. Eng. Data 2010, 55, 4227.

[20] J. W. Goodwin , J. Hearn , C. C. Ho , R. H. Ottewill , Colloid Polym. Sci. 1974, 252, 464.

[21] Y. Chonde , I. M. Krieger , J. Appl. Polym. Sci. 1981, 26, 1819.

[22] H. Kim , Y. Cang , E. Kang , B. Graczykowski , M. Secchi , M. Montagna , R. D. Priestley , E. M. Furst , G. Fytas , Nat. Commun. 2018, 9, 2918. 30046038 10.1038/s41467-018-04854-w PMC6060150

[23] T. Vasileiadis , M. Schöttle , M. Theis , M. Retsch , G. Fytas , B. Graczykowski , Small Methods 2024, 2400855. 10.1002/smtd.202400855 PMC11926515 39139008

[24] M. Montagna , Phys. Rev. B 2008, 77, 045418.

[25] T. Still , High Frequency Acoustics in Colloid‐Based Meso‐ and Nanostructures by Spontaneous Brillouin Light Scattering, Springer, Berlin, Heidelberg 2010.

[26] T. Still , M. Mattarelli , D. Kiefer , G. Fytas , M. Montagna , J. Phys. Chem. Lett. 2010, 1, 2440.

[27] J. N. Israelachvili , Intermolecular and Surface Forces, 3rd ed., Academic Press, Burlington, MA 2011.

[28] K. L. Johnson , K. Kendall , A. D. Roberts , Proc. R. Soc. Math. Phys. Eng. Sci. 1971, 324, 301.

[29] J. N. Israelachvili , Intermolecular and Surface Forces, Elsevier, Amsterdam 2011, pp. 415–467.

[30] M. Mattarelli , M. Montagna , T. Still , D. Schneider , G. Fytas , Soft Matter 2012, 8, 4235.

[31] W. R. Vieth , P. M. Tam , A. S. Michaels , J. Colloid Interface Sci. 1966, 22, 360.

[32] R. Battino , H. L. Clever , Chem. Rev. 1966, 66, 395.

[33] S. Garde , A. E. García , L. R. Pratt , G. Hummer , Biophys. Chem. 1999, 78, 21. 17030303 10.1016/s0301-4622(99)00018-6

[34] Y. Sato , M. Yurugi , K. Fujiwara , S. Takishima , H. Masuoka , Fluid Phase Equilib. 1996, 125, 129.

[35] H. Lamb , Proc. Lond. Math. Soc. 1881, 1, 189.

[36] D. S. Hughes , J. L. Kelly , Phys. Rev. 1953, 92, 1145.

[37] A. Ghanem , M. Khanolkar , A. Wallen , S. P. Helwig , M. Hiraiwa , M. Maznev , A. A. Vogel , N. Boechler , N. Longitudinal , Nanoscale 2019, 11, 5655. 30865190 10.1039/c8nr08453j

[38] M. Rey , T. Yu , R. Guenther , K. Bley , N. Vogel , Langmuir 2019, 35, 95. 30543431 10.1021/acs.langmuir.8b02605

