# Supplementary material for: Surface Engineering of Polymeric Colloidal Crystals by Temperature – Pressure Annealing
Source: Macromol Rapid Commun. 2024 Nov 5;45(24):2400668. doi: 10.1002/marc.202400668 (PMC11661671; doi:10.1002/marc.202400668)
Supplement: Supplementary file 1 — Supporting Information [file MARC-45-2400668-s001.docx]

**Supporting Information**

**Surface engineering of polymeric colloidal crystals by**

**temperature - pressure annealing**

Jeena Varghese^1^, Visnja Babacic^1^, Mikolaj Pochylski^1^, Jacek Gapinski^1^, Hans-Juergen Butt^2^, George Fytas^2,3^*, Bartlomiej Graczykowski^1,2^*

^1^ Faculty of Physics, Adam Mickiewicz University, Uniwersytetu Poznanskiego 2,

61-614 Poznan, Poland.

^2^ Max Planck Institute for Polymer Research, Ackermannweg 10, 55128 Mainz, Germany.

^3^Institute of Electronic Structure and Laser, F.O.R.T.H, N. Plastira 100,70013, Heraklion, Greece.

***E-mail:** [**fytas@mpip-mainz.mpg.de**](mailto:fytas@mpip-mainz.mpg.de)**,** [**graczykowski@mpip-mainz.mpg.de**](mailto:graczykowski@mpip-mainz.mpg.de)

**Materials**

**Symbols**

$d$ (nm) – particle diameter

$R$ (nm) – particle radius

$p$ (bar) – gas pressure

$T$ (K) – Temperature

$T_{g}$ (K) – glass transition temperature

$T_{s}$(K) – softening temperature

$f$(GHz) – frequency of vibration

$\lambda$ (nm) – wavelength of the laser

$v_{l}$ (ms^-1^) – longitudinal sound velocity

$v_{t}$ (ms^-1^) – transverse sound velocity

$E_{PS}$ (GPa) – Young modulus of bulk PS

$E_{\mathrm{eff}}$ (GPa) – Effective elastic constant

$\nu$ – Poisson ratio

$\rho$ (kg m^-3^) – mass density

$M$ (kg) – mass of the particle

$K$ – stiffness constant

$W_{PS-PS}$(J m^-2^) – work of adhesion between two PS particles.

$a_{0}$ (nm) – contact radius

**Table S1.:** Material properties of polystyrene (PS).^1–4^

| $d$ | $v_{t}$ | $v_{l}$ | $E_{PS}$ | $\nu$ | $\rho$ | $W_{PS-PS}$ | $E_{eff (bulk PS)}$ | $T_{s}$ | $T_{g}$ |
| --- | --- | --- | --- | --- | --- | --- | --- | --- | --- |
| 268 | 1210 | 2350 | 4.1 | 0.32 | 1050 | 0.0636 | 5.79 | 343 | 375 |


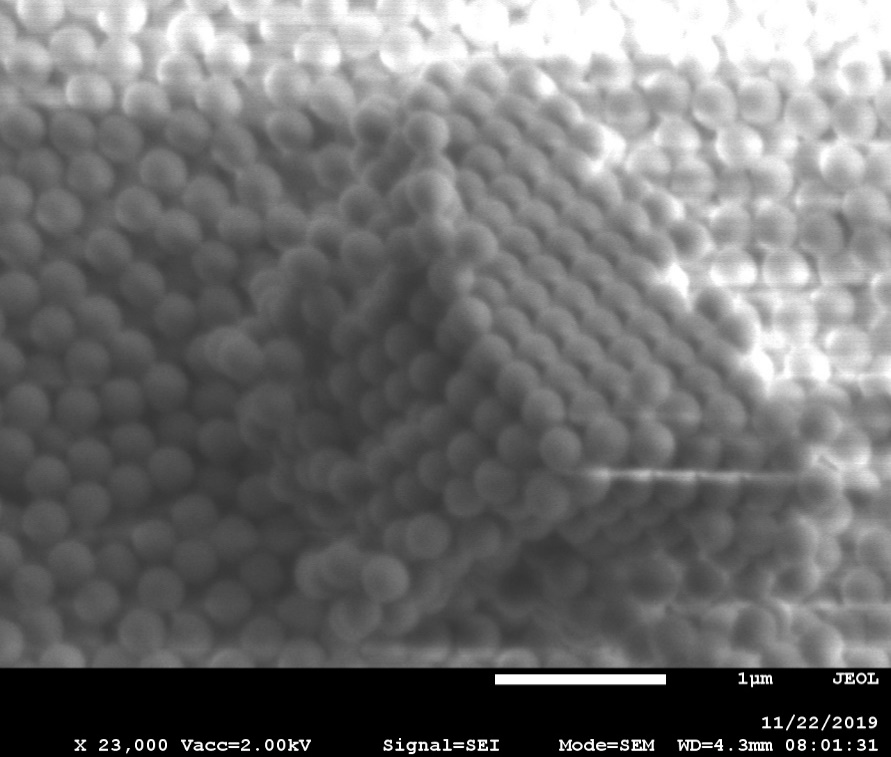


**Fig S1:** SEM image of as-prepared (3D, drop casted) PS268 CC showing the crystalline arrangement of particles.

**Table S2.:** The frequency values of the peaks corresponding to dipolar (1,1) and quadrupolar (1,2) spheroidal modes obtained for PS 268 CCs at different experimental conditions, as described in Figure 1.

| $[T,P]$ conditions | $f_{1,1}$  GHz | $f_{1}$  GHz | $f_{2}$  GHz | $f_{1,2}$  GHz |
| --- | --- | --- | --- | --- |
| RT (296 K), 1 bar | 1.61$\pm$ 0.04 | 3.95$\pm$0.01 | 4.28 $\pm$0.01 | 3.63$\pm$0.01 |
| 296 K, 1000 bar N_2_ | 2.35$\pm$0.02 | 4.35$\pm$0.03 | 4.83$\pm$0.13 | 3.88 $\pm$0.15 |
| 296 K, 1 bar air, –  after $p$treatment | 1.70$\pm$0.04 | 3.96$\pm$0.01 | 4.32$\pm$0.01 | 3.61 $\pm$0.01 |
| 335 K, 1 bar air | 1.65$\pm$0.01 | 3.85$\pm$0.01 | 4.17$\pm$0.01 | 3.53 $\pm$ 0.01 |
| 335 K, 1000 bar N_2_ | 2.82$\pm$0.07 | 4.14 $\pm$ 0.02 | 4.66$\pm$ 0.03 | 3.62$\pm$ 0.05 |
| 335 K, 1 bar air, –  after $p$treatment | 2.49 $\pm$0.01 | 3.97$\pm$ 0.01 | 4.57 $\pm$ 0.02 | 3.37$\pm$ 0.02 |


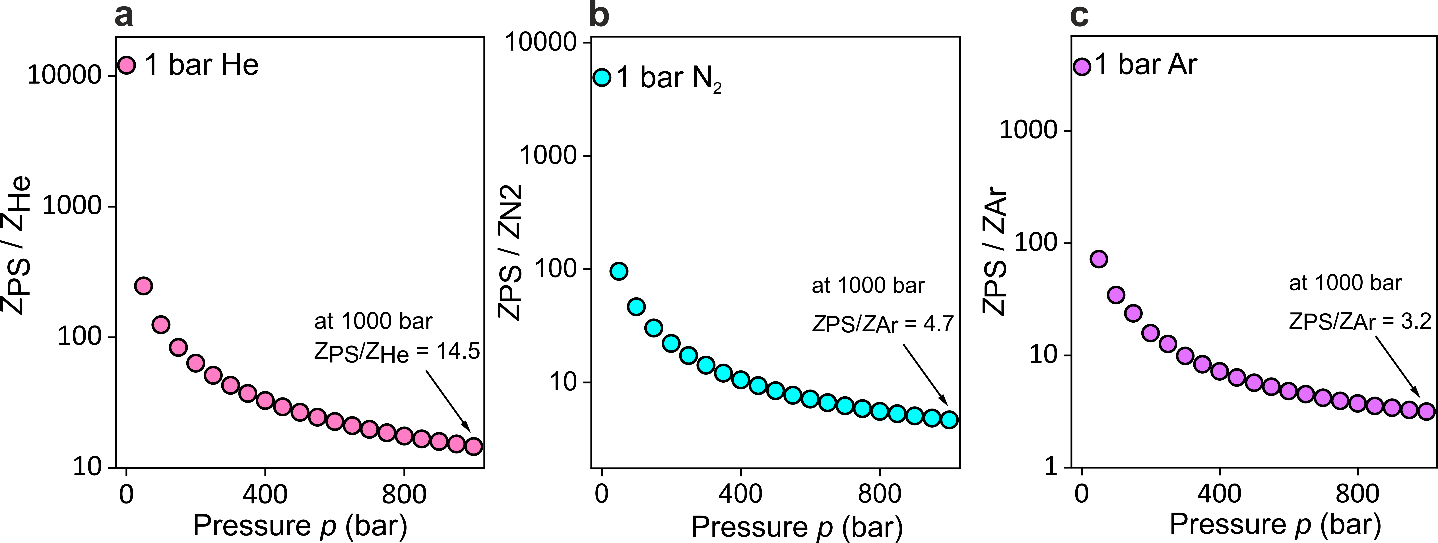


**Fig. S2:** Acoustic impedance contrast between PS and N_2_ as a function of N_2_ gas pressure. Measurements and calculations as described in ref. ^5^.

**Table S3:** Effective elastic constants after $[T,p]$ annealing.

$E_{\mathrm{eff}}$ (pristine) = 0.7 GPa

| $P$ annealing at constant $T$ vales (K) | $E_{\mathrm{eff}}$before  $p$ annealing (GPa) | $E_{\mathrm{eff}}$after  $p$ annealing (GPa) | $\frac{E_{\mathrm{eff}}}{E_{eff(1 bar air, T (before pressure)}}$ | $\frac{E_{\mathrm{eff}}}{E_{\mathrm{bulk}}}$ |
| --- | --- | --- | --- | --- |
| 296 | **0.70** $\boldsymbol{\pm}$ **0.03** | 0.80 $\pm$ 0.04 | 1.12 | 0.14 |
| 309.5 | 0.690 $\pm$ 0.003 | 0.878 $\pm$ 0.003 | 1.27 | 0.15 |
| 323 | 0.55 $\pm$ 0.06 | 1.22 $\pm$0.04 | 2.21 | 0.21 |
| 335.5 | 0.747 $\pm$ 0.005 | 1.734 $\pm$ 0.007 | 2.32 | 0.299 |
| 348 | 0.91 $\pm$ 0.04 | 1.72 $\pm$ 0.01 | 1.89 | 0.297 |





**Fig. S3:** Normalized frequency $f_{12}^{*}\left( p \right)=$ $f_{12}(p)/f_{12}(1 bar, RT)$ of the quadrupolar (1,2) mode as a function of N_2_ pressure increase (purple solid circles) and decrease (hollow circles) measured for PS 268 CGs at specific temperatures, 296 K (RT) (**a**), 335.5 K (**b**), 348 K (**c**). Dashed green lines in (a - c) represent the trend calculated considering only the nonlinear stiffening of polystyrene with pressure increase.

**Calculation of contact radius from BLS measurements**

Using the phonon density of states (DOS) of an *fcc* crystal, the frequency of the (1,1) mode $f_{11}$ can be related to the effective stiffness constant $K_{\mathrm{eff}}$ of the interparticle contacts given by:

| $2\pi f_{11}=2\left( \frac{2K_{\mathrm{eff}}}{M} \right)^{\frac{1}{2}}$, | (1) |
| --- | --- |

, where $M$ is the mass of the NP, $M=\frac{\pi}{6}d^{3}\rho$ and the effective stiffness constant ($K_{\mathrm{eff}}$) can be expressed as ^6^:

|  | $K_{\mathrm{eff}}=\frac{9}{5}\left( \frac{3\pi W_{PS-PS}{R_{\mathrm{eff}}^{2}E}_{PS(eff)}^{2}}{4} \right)^{\frac{1}{3}},$ | (2) |
| --- | --- | --- |

where $W_{PS-PS}$ is the work of adhesion between two PS spheres, the effective radius $R_{\mathrm{eff}}$is calculated from the expression:

|  | $\frac{1}{R_{\mathrm{eff}}}=\frac{1}{R_{1}}+\frac{1}{R_{2}}$ | (3) |
| --- | --- | --- |

and for monodispersed NPs, $R_{1}=$ $R_{2}=R; R_{\mathrm{eff}}=\frac{R}{2}$ .

The effective Young modulus $E_{PS(eff)}$ of two PS spheres is given by:

|  | $E_{PS(eff)}=\frac{4}{3}\left( \frac{1-\nu_{1}^{2}}{E_{PS1}}+\frac{1-\nu_{2}^{2}}{E_{PS2}} \right)^{-1}$ | (4) |
| --- | --- | --- |

Using the bulk properties of PS listed in table S1 in SI, $\nu$ = 0.32, $E_{\mathrm{PS}}$= 4.1, for two identical PS NPs, $E_{PS(eff)}$ can be simplified to:

|  | $E_{PS(eff)}=\frac{2}{3}\frac{E_{\mathrm{PS}}}{(1-\nu^{2})}=$ 3.05 GPa | (5) |
| --- | --- | --- |

From Johnson-Kendall-Roberts (JKR) model ^6–8^, under zero load, the contact radius ($a_{0}^{\mathrm{JKR}}$) between two identical NPs is given by:

| $a_{0}^{\mathrm{JKR}}=\left( \frac{3\pi d^{2}W_{PS-PS}}{8E_{PS(eff)}} \right)^{\frac{1}{3}}$ | (6) |
| --- | --- |

Using Eqns. 1 - 6, we can relate $f_{11}$ to $a_{0}^{\mathrm{JKR}}$ by:

| $a_{0}^{\mathrm{JKR}}=\frac{5\pi^{2}Mf_{11}^{2}}{9E_{PS(eff)}}$ | (7) |
| --- | --- |

**Resilience test to determine the mechanical robustness of [**$\boldsymbol{T,p]}$ **annealed PCCs**

The polymer colloidal crystalline (PCC) samples are fabricated by drop casting of aqueous suspensions on a glass slide and subsequent drying at room temperature under vacuum. The crack formation after the drying process is evident in the as-prepared samples in Fig. S4 a,b. We conducted a resilience test to determine the mechanical robustness of the dry colloidal crystals after the temperature-pressure annealing. The as-prepared sample in Fig. 1b is subjected to [$T,p]$ annealing at 338 K and 400 bar of Ar gas pressure for 3h. The resilience test is done by hitting the lateral sides of the glass slide on the tabletop. We can observe that the as-prepared sample is entirely detached from the substrate (Fig 1c). However, the [$T,p]$annealed sample (Fig. 1d) survives the external stress to a great extent. Note that some fraction of PS CC is detached (inset of Fig. 1 d) before the resilience test. Crack formation during the fabrication of CCs cannot be avoided. However, the mechanical robustness of the fragile CCs is significantly improved by [$T,p]$annealing ^4^.


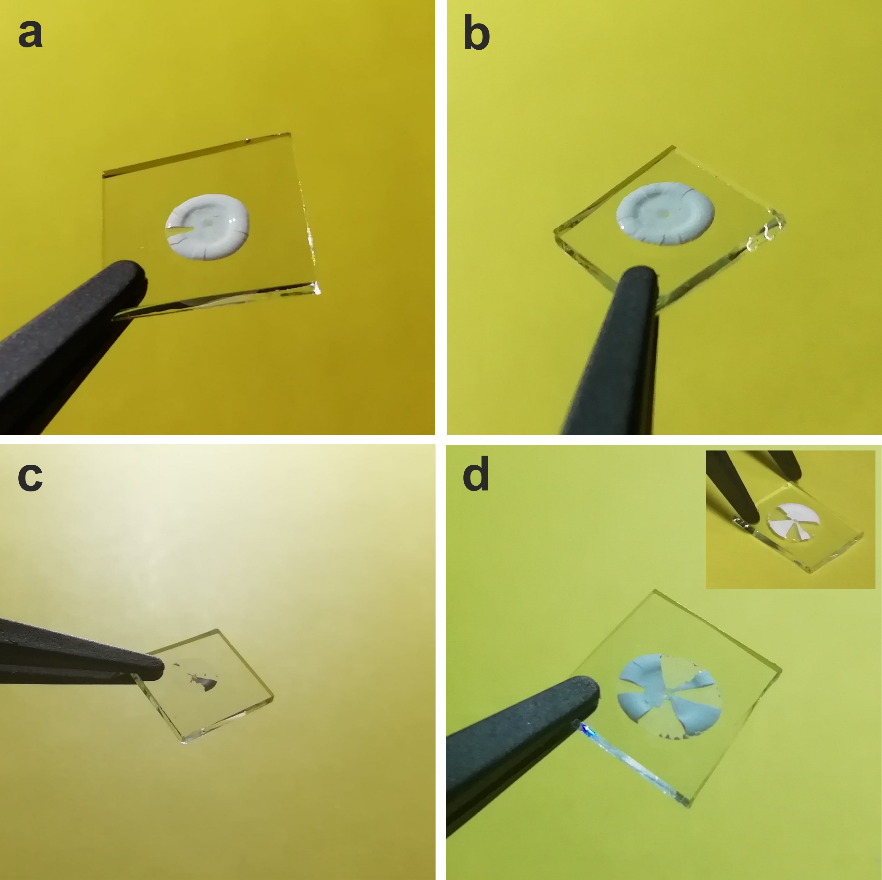


**Fig. S4:** Fabrication of polymer colloidal crystalline (PCC) films prepared by drop casting of an aqueous dispersion of PS NPs onto a glass substrate (a, b). As-prepared PCC sample (c) and sample that was treated at 338 K and 400 bar of Ar for 3h (d) after the resilience test. Inset in (d) shows the PCC sample after [$T,p]$ annealing. Reproduced with permissions from (ref. 4): V. Babacic, J. Varghese et al. ; *J. Colloid Interface Sci.* **2020**, *579*, 786–793. https://doi.org/10.1016/j.jcis.2020.06.104.

**References:**

(1) Kim, H.; Cang, Y.; Kang, E.; Graczykowski, B.; Secchi, M.; Montagna, M.; Priestley, R. D.; Furst, E. M.; Fytas, G. Direct Observation of Polymer Surface Mobility via Nanoparticle Vibrations. *Nat. Commun.* **2018**, *9* (1), 2918.

(2) Graczykowski, B.; Vogel, N.; Bley, K.; Butt, H.-J.; Fytas, G. Multiband Hypersound Filtering in Two-Dimensional Colloidal Crystals: Adhesion, Resonances, and Periodicity. *Nano Lett.* **2020**, *20* (3), 1883–1889.

(3) Hughes, D. S.; Kelly, J. L. Second-Order Elastic Deformation of Solids. *Phys. Rev.* **1953**, *92* (5), 1145–1149. https://doi.org/10.1103/PhysRev.92.1145.

(4) Babacic, V.; Varghese, J.; Coy, E.; Kang, E.; Pochylski, M.; Gapinski, J.; Fytas, G.; Graczykowski, B. Mechanical Reinforcement of Polymer Colloidal Crystals by Supercritical Fluids. *J. Colloid Interface Sci.* **2020**, *579*, 786–793. https://doi.org/10.1016/j.jcis.2020.06.104.

(5) Varghese, J.; Mohammadi, R.; Pochylski, M.; Babacic, V.; Gapinski, J.; Vogel, N.; Butt, H.-J.; Fytas, G.; Graczykowski, B. Size-Dependent Nanoscale Soldering of Polystyrene Colloidal Crystals by Supercritical Fluids. *J. Colloid Interface Sci.* **2023**, *633*, 314–322. https://doi.org/10.1016/j.jcis.2022.11.090.

(6) Johnson, K. L.; Kendall, K.; Roberts, A. D. Surface Energy and the Contact of Elastic Solids. *Proc. R. Soc. Math. Phys. Eng. Sci.* **1971**, *324* (1558), 301–313. https://doi.org/10.1098/rspa.1971.0141.

(7) Israelachvili, J. N. *Intermolecular and Surface Forces*, 3rd ed.; Academic Press: Burlington, MA, 2011.

(8) Israelachvili, J. N. Adhesion and Wetting Phenomena. In *Intermolecular and Surface Forces*; Elsevier, 2011; pp 415–467. https://doi.org/10.1016/B978-0-12-375182-9.10017-X.
